# Anemia and perioperative mortality in non-cardiac surgery patients: a secondary analysis based on a single-center retrospective study

**DOI:** 10.1186/s12871-020-01024-8

**Published:** 2020-05-11

**Authors:** Xueying Luo, Feng Li, Haofei Hu, Baoer Liu, Sujing Zheng, Liping Yang, Rui Gao, Ya Li, Rao Xi, Jinsong He

**Affiliations:** 1 Department of Plastic and reconstructive, Shenzhen People’s Hospital, No. 1017, Dongmen North Road, Luohu District, Shenzhen, ,518000 Guangdong China; 2 Department of Breast thyroid surgery, Shenzhen Breast Cancer Research and Treatment Research Center, Peking University Shenzhen Hospital, Shenzhen, China; 3 Department of Breast thyroid surgery, Shenzhen Breast Cancer Research and Treatment Research Center, Peking University Shenzhen Hospital, Shenzhen, China; 4 Department of Breast thyroid surgery, Shenzhen University, No. 3688 Nanhai Avenue, Nanshan District, Shenzhen, 518000 Guangdong China; 5 Department of Thyroid and Breast surgery, Shenzhen Second People’s Hospital, No. 3002, Sungang West Road, Futian District, Shenzhen, Shenzhen, 518000 Guangdong China; 6 Department of General Medicine, Shenzhen University, No. 3002, Sungang West Road, Futian District, Shenzhen, 518000 Guangdong China; 7 Department of Radiation Oncology, Faculty of Medicine, Universitatsklinikum Freiburg, Freiburg, Germany; 8 Department of Breast thyroid surgery, Shenzhen Breast Cancer Research and Treatment Research Center, Peking University Shenzhen Hospital, 1120 Lianhua Road, Futian District, Shenzhen, 518000 Guangdong China

**Keywords:** anemia, postoperative 30day mortality, non-cardiac surgery, ICU admission, postoperative transfusion, perioperative prognosis

## Abstract

**Background:**

Evidence regarding the relationship between anemia and perioperative prognosis is controversial. The study was conducted to highlight the specific relationship between anemia and perioperative mortality in non-cardiac surgery patients over 18 years of age.

**Methods:**

This study was a retrospective analysis of the electronic medical records of 90,784 patients at the Singapore General Hospital from January 1, 2012 to October 31, 2016. Multivariate regression, propensity score analysis, doubly robust estimation, and an inverse probability-weighting model was used to ensure the robustness of our findings.

**Results:**

We identified 85,989 patients, of whom75, 163 had none or mild anemia (Hemoglobin>90g/L) and 10,826 had moderate or severe anemia (Hemoglobin≤90g/L). 8,857 patients in each study exposure group had similar propensity scores and were included in the analyses. In the doubly robust model, postoperative 30-day mortality rate was increased by 0.51% (*n* = 219) in moderate or severe anemia group (Odds Ratio, 1.510; 95% Confidence Interval (CI), 1.049 to 2.174) compared with none or mild anemia group (2.47% *vs.*1.22%, *P*<0.001). Moderate or severe anemia was also associated with increased postoperative blood transfusion rates (OR, 5.608; 95% CI, 4.026 to 7.811, *P* < 0.001). There was no statistical difference in Intensive Care Unit (ICU) admission rate among different anemia groups within 30 days after surgery (*P*=0.104).

**Discussion:**

In patients undergoing non-cardiac surgery over 18 years old, moderate or severe preoperative anemia would increase the occurrence of postoperative blood transfusion and the risk of death, rather than ICU admission within 30 days after surgery.

## Background

Preoperative anemia affects 30-40% of patients undergoing major surgery and is an independent risk factor for postoperative complications and long-term mortality [1]. However, there is controversy of the relationship between anemia and perioperative prognosis, such as postoperative 30day mortality. It has been reported that the relationship between them is no statistically significant in patients undergoing rectal cancer surgery [2], cardiac surgery [3], hepatectomy [4], single-level lumbar surgery [4]. And conversely, some studies pointing out that anemia is an important predictor of 30day mortality in the patients undergoing, cardiovascular [5–7], spine tumors [8], major abdominal [9] , joint arthroplasty [10], gastrointestinal surgery [11], vascular surgery [12], and thyroidectomy [13]. Little is known about the effects of anemia in the perioperative prognosis in non-cardiac surgery patients over 18 years of age, with two related studies involving children [14] and the elderly [15]. Our study aimed to investigate the relationship between different anemia status and perioperative prognosis in non-cardiac surgery adult patients.

## Methods

### Study design and setting

This study was a secondary analysis based on a single-center retrospective study, that had been conducted a single-center retrospective study from January 1, 2012 to October 31, 2016 at the Singapore General Hospital. In the present study, it was performed to address the relationship between anemia status and perioperative prognosis. The target independent variable is anemia status obtained at baseline.

### Participants and Procedures

Patients who underwent cardiac surgery, burn-related surgery, neurosurgery, and transplantation were excluded due to their categorically higher mortality rate and blood transfusion requirement, based on the original research. A total of 90785 surgical patients were recruited and selected for the study. Only surgical patients, over 18 years of age, with complete anemia data can qualified for inclusion in the study.

Covariates included in this study were specified a priori as potential confounders on the relationship of anemia and perioperative prognosis in patients, based on clinical experience and previous studies. The data collected during the preoperative anesthetic assessment visit included age, gender, race, preoperative estimated glomerular filtration rate (eGFR),presence of cerebrovascular accidents (CVA), diabetes mellitus (DM),ischemic heart disease (IHD),congestive heart failure (CHF),red cell distribution (RDW), priority of surgery, anesthesia type, surgical risk, preoperative blood transfusion with in 30days, intraoperative blood transfusion data, the Revised Cardiac Risk Index (RCRI) score, the ASA status. Preoperative laboratory results including renal group (including eGFR) and full blood count (including hemoglobin concentration and RDW) were taken as the latest blood results within 90 days before surgery, and up to the day of surgery. RDW is the coefficient of variation (percentage) between the red blood cell volume and the normal reference range of RDW, ranging from 10.9% to 15.7%. Levels >15.7% were defined as high RDW. The severity of anemia was defined by WHO’s gender-based classification of hemoglobin concentration. Mild anemia was defined as hemoglobin concentration of 11–12.9g/dL in males and 11–11.9g/dL in females; moderate anemia was defined for both genders to be hemoglobin concentration between 8–10.9g/dL and severe anemia defined as hemoglobin concentration <8.0g/dL. Priority of surgery (emergency or elective) and surgical risk classification were based on the 2014 European Society of Cardiology (ESC) and the European Society of Anaesthesiology (ESA) guidelines [16, 17]. American Society of Anesthesiologists-Physical Status (ASA-PS) follows that of the ASA-PS definitions [17].

The patients were followed up for 30 days after their index operation to identify all ICU admissions (stay time >24 hours), blood transfusion and mortality. Mortality data (the primary outcome) were synchronized with the National Electronic Health Records, ensuring a near complete follow-up [18]. The need for ICU stay (>24 hours) during surgical admission may serve as a surrogate marker for major postoperative complications.

### Dataset

We downloaded the raw data for free from the DATADRYAD database (www.datadryad.org). Since Diana Xin Hui Chan et al. transferred the ownership of the original data to the DATADRYAD website, we were able to use this data for secondary data analysis based on different scientific assumptions (Dryad data package: Chan, Diana Xin Hui et al. (2018), Data from: Development of the Combined Assessment of Risk Encountered in Surgery (CARES) surgical risk calculator for prediction of post-surgical mortality and need for intensive care unit admission risk – a single-center retrospective study, Dryad, Dataset, 10.5061/dryad.v142481). Since our study was based on a secondary analysis of past data and the patient's personal information in the original data was anonymous, there was no need for informed consent from the participants. The ethical approval was described in the published paper [19].

### Statistical analysis

Considering the differences in baseline characteristics between the two groups of eligible participants (Table 1), propensity score matching was used to identify a cohort of patients with similar baseline characteristics. Matching was performed with the use of a 1:1 matching protocol without replacement (greedy-matching algorithm), with a caliper width equal to 0.05. Covariate balances before and after PS matching was assessed using standardized differences. For a given covariate, standardized differences of less than 10.0%indicate a relatively small imbalance.

**Table 1:** Baseline characteristics of participants

	FULL COHORT (*N* =85 989)			Propensity Score–Matched Cohort (*n* = 17 714)		
ANEMIA CATEGORY	NONE OR MILD	MODERATE OR SEVERE	SD (100%)	NONE OR MILD	MODERATE OR SEVERE	SD (100%)
N	75163	10826		8857	8857	
AGE (years)	52.456 ± 16.456	58.142 ± 17.295	33.7%	60.41 ± 15.94	59.23 ± 16.49	7.0%
sex			33.2%			9.0%
Male	35907 (47.772%)	3435 (31.729%)		2672 (30.17%)	3061 (34.56%)	
Female	39256 (52.228%)	7391 (68.271%)		6185 (69.83%)	5796 (65.44%)	
RACE			14.3%			10.0%
Chinese	54347 (72.309%)	7448 (68.797%)		6267 (70.76%)	6247 (70.53%)	
Indian	6570 (8.741%)	976 (9.015%)		666 (7.52%)	774 (8.74%)	
Malay	7014 (9.332%)	1489 (13.754%)		1002 (11.31%)	1132 (12.78%)	
Others	7228 (9.617%)	913 (8.433%)		922 (10.41%)	704 (7.95%)	
PREOP-EGFR	69.304-104.537	27.641-106.590	26.5%	42.39-98.44	28.36-107.44	3.0%
RDW N (%)			99.4%			56.0%
RDW≤15.7	69383 (92.310%)	6013 (55.542%)		7373 (83.24%)	5263 (59.42%)	
RDW>15.7	3765 (5.009%)	4659 (43.035%)		1426 (16.10%)	3569 (40.30%)	
NA	2015 (2.681%)	154 (1.423%)		58 (0.65%)	25 (0.28%)	
Preop-transfusion with in 30days n(%)			41.9%			3.0%
0 units	74439 (99.037%)	9690 (89.507%)		8247 (93.11%)	8208 (92.67%)	
1 unit	410 (0.545%)	597 (5.515%)		340 (3.84%)	339 (3.83%)	
2 or more units	314 (0.418%)	539 (4.979%)		270 (3.05%)	310 (3.50%)	
Intraop-transfusion			68.1%			2.0%
0 units	72917 (97.012%)	8063 (74.478%)		7105 (80.22%)	7170 (80.95%)	
1 unit	2246 (2.988%)	2763 (25.522%)		1752 (19.78%)	1687 (19.05%)	
CVA CATEGORY			13.1%			2.0%
NO	51068 (67.943%)	7133 (65.888%)		5819 (65.70%)	5864 (66.21%)	
YES	1142 (1.519%)	386 (3.565%)		302 (3.41%)	325 (3.67%)	
NA	22953 (30.538%)	3307 (30.547%)		2736 (30.89%)	2668 (30.12%)	
CHF CATEGORY			17.2%			4.0%
NO	53543 (71.236%)	7415 (68.493%)		6114 (69.03%)	6132 (69.23%)	
YES	475 (0.632%)	308 (2.845%)		191 (2.16%)	247 (2.79%)	
NA	21145 (28.132%)	3103 (28.662%)		2552 (28.81%)	2478 (27.98%)	
IHD CATEGORY			22.9%			4.0%
NO	48887 (65.041%)	6416 (59.265%)		5248 (59.25%)	5252 (59.30%)	
YES	3128 (4.162%)	1068 (9.865%)		816 (9.21%)	909 (10.26%)	
NA	23148 (30.797%)	3342 (30.870%)		2793 (31.53%)	2696 (30.44%)	
DM CATEGORY			25.7%			13.0%
NO	52257 (69.525%)	6909 (63.819%)		5868 (66.25%)	5711 (64.48%)	
YES	1262 (1.679%)	723 (6.678%)		342 (3.86%)	607 (6.85%)	
NA	21644 (28.796%)	3194 (29.503%)		2647 (29.89%)	2539 (28.67%)	
Anesthesia type n(%)			17.7%			5.0%
ga	63448 (84.414%)	8389 (77.489%)		6805 (76.83%)	6989 (78.91%)	
ra	11715 (15.586%)	2437 (22.511%)		2052 (23.17%)	1868 (21.09%)	
Priority of surgery n(%)			33.2%			8.0%
Elective	60799 (80.890%)	7197 (66.479%)		5809 (65.59%)	6160 (69.55%)	
Emergency	14364 (19.110%)	3629 (33.521%)		3048 (34.41%)	2697 (30.45%)	
Surgical risk			26.7%			7.0%
Low	39779 (52.924%)	4531 (41.853%)		3599 (40.63%)	3880 (43.81%)	
Moderate	32691 (43.493%)	5417 (50.037%)		4612 (52.07%)	4314 (48.71%)	
High	2693 (3.583%)	878 (8.110%)		646 (7.29%)	663 (7.49%)	
RCRI CATEGORY			52.7%			18.0%
I	41157 (54.757%)	3769 (34.814%)		3484 (39.34%)	3138 (35.43%)	
II	9884 (13.150%)	2516 (23.240%)		1766 (19.94%)	2066 (23.33%)	
III	1559 (2.074%)	869 (8.027%)		476 (5.37%)	728 (8.22%)	
IV	473 (0.629%)	407 (3.759%)		224 (2.53%)	336 (3.79%)	
NA	22090 (29.389%)	3265 (30.159%)		2907 (32.82%)	2589 (29.23%)	
ASA CATEGORY			72.3%			35.0%
1	18716 (24.901%)	1109 (10.244%)		1117 (12.61%)	816 (9.21%)	
2	43132 (57.385%)	4592 (42.416%)		4447 (50.21%)	3839 (43.34%)	
3	9813 (13.056%)	4453 (41.132%)		2379 (26.86%)	3738 (42.20%)	
NA	3502 (4.659%)	672 (6.207%)		914 (10.32%)	464 (5.24%)	
Postop- transfusion			40.7%			28.0%
0 units	75081 (99.891%)	9966 (92.056%)		8787 (99.21%)	8352 (94.30%)	
≥1 unit	82 (0.109%)	860 (7.944%)		70 (0.79%)	505 (5.70%)	
ICUADMGT24H			19.1%			00.0%
No	74366 (98.940%)	10386 (95.936%)		8542 (96.44%)	8542 (96.44%)	
Yes	797 (1.060%)	440 (4.064%)		315 (3.56%)	315 (3.56%)	
THIRTY-DAY MORTALITY N(%)			21.4%			09.0%
No	74955 (99.723%)	10505 (97.035%)		8749 (98.78%)	8638 (97.53%)	
Yes	208 (0.277%)	321 (2.965%)		108 (1.22%)	219 (2.47%)	

Noted: SD was calculated by Kruskal-Wallis H test

Abbreviations: *GA* general anesthesia, *RA* regional anesthesia, *PREOP-eGFR* preoperative estimated glomerular filtration rate (mL/min/1.73m2), *RDW* red cell distribution, *NA* not available, *CVA* cerebrovascular accidents, *IHD* ischemic heart disease, *CHF* congestive heart failure, *DM* diabetes mellitus requiring insulin therapy; creatinine>2.0mg/dl, *Preop* preoperative, *Intraop* intraoperative, *Postop* postoperative, *RCRI* Revised Cardiac Risk Index, *ASA* American Society of Anesthesiologists, *ICU* Intensive Care Unit, *ICUADMGT24H* admission to ICU for >24 hours

The doubly robust estimation method, the combination of multivariate regression model and a propensity score model, was also applied to infer the independent associations between anemia status and patients’ primary and secondary outcomes [20, 21]. Using the estimated propensity scores as weights, an inverse probabilities weighting (IPW) model was used to generate a weighted cohort [22] . A logistic regression was then performed on the weighted cohort, adjusting for the variables that remained unbalanced between different anemia groups in the propensity score model.

### Sensitivity analysis

We conducted a series of sensitivity analyses to evaluate the robustness of the findings of the study and how our conclusions can be affected by applying various association inference models. In the sensitivity analysis, we applied three more association inference models: a propensity score-based IPW model, a propensity score-based patient-matching model, and a logistic regression-based multivariate analysis model. The calculated effect sizes and p values from all these models were reported and compared.

Continuous variables were expressed as mean ± standard deviation (normal distribution) or median (interquartile range) (skewed distribution), and categorical variables were expressed in frequency or as a percentage. In the process of multivariate regression analysis, there are some confounders with partial missing data. If it is a categorical variable, the missing data would be directly treated as a new independent group; if it is a continues variable, the missing data would be replaced with an average or median value. The T test (normal distribution), Mann-Whitney (skewed distribution) tests and chi-square tests (categorical variables) were used to determine any statistical differences between the means and proportions of the anemia groups. All of the analyses were performed with the statistical software packages R (http://www.R-project.org, The R Foundation) and EmpowerStats (http://www.empowerstats.com, X&Y Solutions, Inc., Boston, MA). P values less than 0.05 (two-sided) were considered statistically significant.

## Results

### The selection of participants

**Fig. 1:**
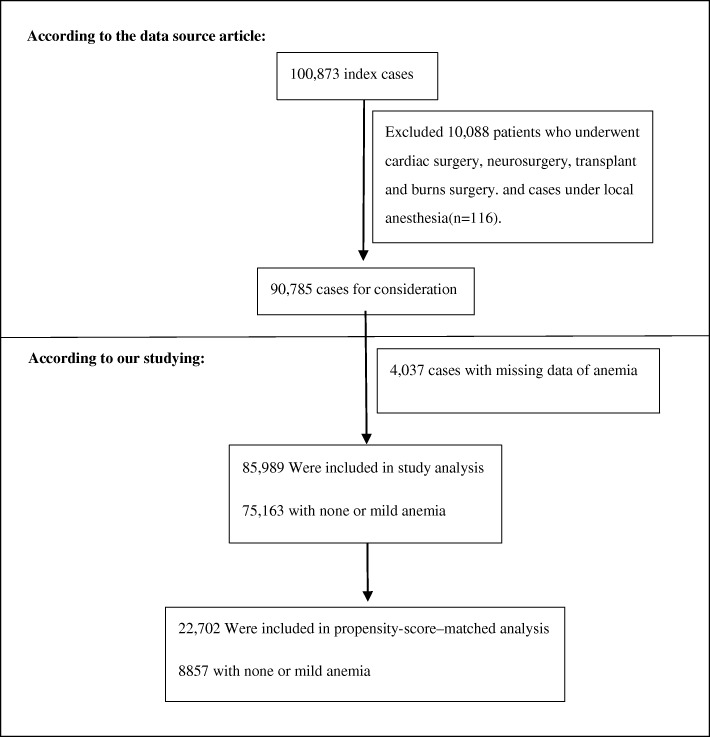
Study Population

After excluding 4,037 cases with missing data of anemia status and 758 cases under 18 years of age, the study's initial cohort was recruited the initial cohort for this study was recruited(N = 85 989;mean± age:53.17 ± 16.67 years; 54.25%female ).There were 75,163 (87.4%) patients with none or mild anemia, and 10,826 (12.6%) patients with moderate or severe anemia (Fig. 1).One-to-one propensity score matching yielded 22,702 patients, with 8857 patients in each study exposure group. Patient characteristics were well balanced between exposure groups (Table 1). The standard deviation of almost all variables is less than 10%, indicating that the propensity scores are perfectly matched (Figure S1).

### Baseline characteristics of participants

Prior to the propensity score matching, we found that in the moderate or severe anemia group, patients were usually older, more women, more frequent preoperative and intraoperative blood transfusions, higher RDW, and a higher incidence of comorbidities ,emergency surgery with higher surgical risk (based on ASA, RCRI, and surgical risk assessment). Corresponding postoperative blood transfusion times, ICU admission rates and 30-day mortality were higher. There were substantial differences between the none or mild and moderate or severe anemia groups, which highlights the need to match participants based on confounding factors. After matching at a 1: 1 ratio, we found that the included covariates were well balanced in different anemia groups. In the matching analysis, the RDW, DM, RCRI score, and ASA status are not well balanced. Therefore, we performed additional adjusted regression analysis on these variables.

### Outcomes

We also showed the doubly robust estimation model, propensity score-based IPW model, and propensity score-based patient-matching model of the matched cohort in the results of multivariate analysis, and the logistic regression-based multivariate analysis model before propensity score matching (Table 2 and Table 3). In the double robust estimation model, the risk of moderate or severe anemia and postoperative blood transfusion was significantly higher than that of the group without or with mild anemia (OR=5.608; 95% CI, 4.026 to 7.811; P<0.001) and thirty-day mortality (OR=1.510, 95% CI: 1.049 to 2.174; P=0.027). In the propensity score-based IPW model, similar relationships of moderate or severe anemia with postoperative blood transfusions (OR=7.456, 95% CI: 5.397 to 10.30; *P*<0.001) and thirty-day mortality (OR=1.996, 95% CI: 1.413 to 2.819; *P*<0.001) still existed. The effect values of moderate or severe anemia were similar to those mentioned above in the propensity score-based patient-matching model (Postoperative blood transfusions: OR=8.566, 95% CI:6.571 to 11.17;Thirty-day mortality: OR=1.936, 95% CI: 1.530 to 2.449), and in the logistic regression-based multivariate analysis model (Postoperative blood transfusions :OR=7.187, 95% CI: 5.557 to 9.296; Thirty-day mortality: OR=1.917, 95% CI: 1.531 to 2.400). There was no statistical difference in the admission to ICU within 30 days after surgery between different status of anemia, whether in the doubly robust estimation method (OR=0.810, 95% CI: 0.628 to 1.044; *P*=0.104),the propensity score-based patient-matching model (OR=0.923, 95% CI:0.784 to 1.087; *P*=0.337) ,the propensity score-based IPW model (OR=0.964, 95% CI:0.759 to 1.224; *P*=0.763),and logistic regression-based multivariate analysis model (OR=0.848, 95% CI: 0.714 to 1.008; *P*=0.061).

**Table 2:** The results of univariate and multivariate analyses before propensity score matching

ANEMIA CATEGORY	model i	ModEL iI	ModEL iIi
Postop- transfusion			
None or mild	Ref	Ref	Ref
Moderate or severe	79.01 (62.936, 99.194) <0.001	76.924 (61.075, 96.885) <0.001	7.187 (5.557, 9.296) <0.001
ICUADMGT24H			
None or mild	Ref	Ref	Ref
Moderate or severe	3.953 (3.512, 4.449) <0.001	3.560 (3.146, 4.028) <0.001	0.848 (0.714, 1.008) 0.061
THIRTY-DAY MORTALITY			
None or mild	Ref	Ref	Ref
Moderate or severe	11.011 (9.238, 13.126) <0.001	8.395 (6.989, 10.084) <0.001	1.917 (1.531, 2.400) <0.001

The results were expressed as odds ratio (95%confidence interval) *P*-value

MODEL I (Non-adjusted model): we did not adjust any covariate

MODEL II (Minimally-adjusted model): we only adjusted age, gender and race

MODEL III (Fully-adjusted model): we adjusted age, sex, race, preoperative eGFR, presence of CVA,DM, IHD, CHF, RDW, priority of surgery, anesthesia type, surgical risk, preoperative blood transfusion with in 30days, intraoperative blood transfusion data, the RCRI score,the ASA status.

**Table 3:** The results of univariate and multivariate analyses in propensity score matched cohort

ANEMIA CATEGORY	model i*	ModEL iI*	ModEL iIi*
Postop- transfusion			
None or mild	Ref	Ref	Ref
Moderate or severe	8.566 (6.571, 11.17) <0.001	7.456 (5.397, 10.30) <0.001	5.608 (4.026, 7.811) <0.001
ICUADMGT24H			
None or mild	Ref	Ref	Ref
Moderate or severe	0.923 (0.784, 1.087) 0.337	0.964 (0.759, 1.224) 0.763	0.810 (0.628, 1.044) 0.104
THIRTY-DAY MORTALITY			
None or mild	Ref	Ref	Ref
Moderate or severe	1.936 (1.530, 2.449) <0.001	1.996 (1.413, 2.819) <0.001	1.510 (1.049, 2.174) 0.027

The results were expressed as odds ratio (95%confidence interval) *P*-value

MODEL I* ( The propensity score-based patient-matching model): we adjusted for propensity score

MODEL II* (The propensity score-based IPW model):we did not adjust any covariates with the propensity score-based IPW

MODEL III*(The doubly robust estimation model): we adjusted for DM, RDW,the RCRI score, the ASA status, with the propensity score-based IPW

## Discussion

This study showed that moderate or severe anemia was significantly associated with higher risks of postoperative blood transfusion and 30-day mortality in non-cardiac and non-surgery patients over 18 years of age compared to the none or mild anemia group. There was a non-significant relationship between different anemia status with the admission to ICU (*P*=0.082). This finding was consistent across different statistical analyses including the doubly robust estimation method, the propensity score-based IPW model, the propensity score-based patient-matching model, and the logistic regression-based multivariate analysis model. It revealed that the uncontrolled moderate or severe anemia before surgery would increase the occurrence of postoperative blood transfusion and the risk of death, rather than critical complications within 30 days after surgery.

A consensus has been reached on the impact of anemia on long-term mortality after surgery. However, there is still considerable controversy over the effect of anemia on perioperative mortality. Many reports indicate that although anemia may increase the risk of surgical complications, it has no effect on 30-day mortality. These research groups involved patients undergoing rectal cancer surgery, cardiac surgery [3, 13, 23, 24], hepatectomy [4], single-level lumbar surgery [25]. Others objected to the above points, insisting that anemia is an important predictor of 30day mortality, mainly in patients undergoing cardiovascular surgery [5–7, 12]. However, studies on multidisciplinary surgical populations for non-cardiac surgery are limited. There are two related studies on this surgical population, mainly involving children [14] and the elderly [15] They have confirmed that anemia is an independent risk factor for 30-day mortality in patients of these ages, while studies of other ages are lacking. At the same time, there is currently a lack of research in different ethnic groups. Our study confirmed that there was no statistically significant difference in the effect of anemia on30-day mortality in different races over 18 years of age, highlighting the importance of controlling anemia before surgery.

This study was powered to compare anemia with perioperative prognosis. We use the doubly robust estimation method to minimize baseline differences between the groups, thus limiting the extent of treatment selection bias inherent in a retrospective study. In addition, we conducted a sensitivity analysis to confirm the reliability of the results. And this clinical database offered significant granularity in terms of demographic information, preexisting comorbidities, and risk assessment methods, which are important independent risk factors for morbidity and mortality. The prediction of the risk for postoperative ICU admission is novel and may serve as a surrogate marker for major postoperative complications.

One limitation of this study is based on a secondary analysis of published data, we can’t exclude some residual and/or unmeasured confounding factors that could bias the estimated association (e.g. inflammatory markers and socioeconomic factors) and investigate the relationship between anemia with long-term outcomes. Other limitation is that although the original surgical population included most non-cardiac surgery populations, it discharged high-risk nerves, burns, etc.

## Conclusion

In patients over 18 years of age undergoing non-cardiac surgery, uncontrolled moderate or severe preoperative anemia increases the incidence of postoperative blood transfusions and increases the risk of death, even if no serious complications are added within 30 days of surgery.

## Supplementary information

**Additional file 1: Figure S1.**

## Data Availability

The data was obtained from ‘DATADRYAD’ database (www.Datadryad.org). This website permitted users to freely download the raw data. (Dryad data package: Development of the Combined Assessment of Risk Encountered in Surgery (CARES) surgical risk calculator for prediction of post-surgical mortality and need for intensive care unit admission risk – a single-center retrospective study, Dryad, Dataset, 10.5061/dryad.v142481).

## Abbreviations

CI: Confidence Interval
OR: Odds Ratio
ICU: Intensive Care Unit
eGFR: estimated Glomerular Filtration Rate
CVA: Cerebrovascular accidents
CHF: Congestive Heart Failure
IHD: Ischemic Heart Disease
DM: Diabetes Mellitus
OR: Odds Ratio
CI: Confidence Interval
CKD: Chronic Kidney Disease
CARES: Combined Assessment of Risk Encountered in Surgery
RCRI score: Revised Cardiac Risk Index Score
ASA-PS: American Society of Anesthesiologists-Physical Status
RDW: Red Cell Distribution Width
eHINTS: SingHealth-IHiS Electronic Health Information System
ESC: European Society of Cardiology
WHO: World Health Organization
GAM: Generalized Additive Model
