# Supplementary figures and images for: Anemia and perioperative mortality in non-cardiac surgery patients: a secondary analysis based on a single-center retrospective study
Source: BMC Anesthesiol. 2020 May 11;20:112. doi: 10.1186/s12871-020-01024-8 (PMC7212669; doi:10.1186/s12871-020-01024-8)

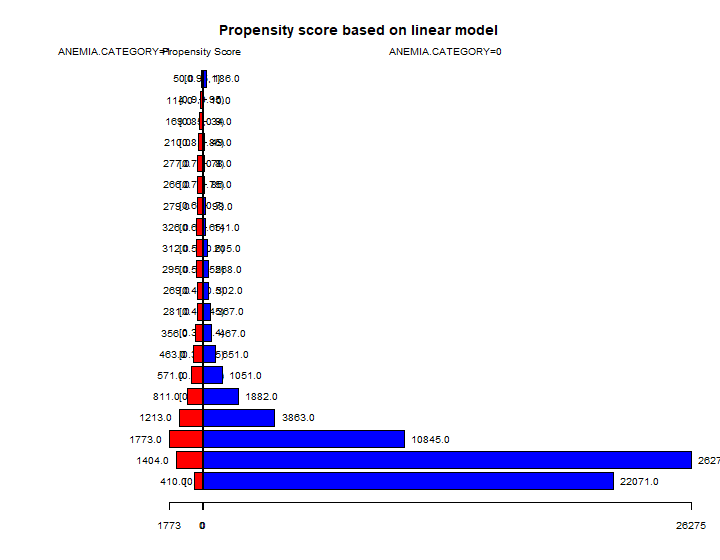

Supplement: Supplementary file 1 — Additional file 1: Figure S1. [file 12871_2020_1024_MOESM1_ESM.png]
